# The CP12 protein family: a thioredoxin-mediated metabolic switch?

**DOI:** 10.3389/fpls.2014.00009

**Published:** 2014-01-30

**Authors:** Patricia E. López-Calcagno, Thomas P. Howard, Christine A. Raines

**Affiliations:** ^1^School of Biological Sciences, University of EssexColchester, UK; ^2^Biosciences, College of Life and Environmental Sciences, University of ExeterExeter, UK

**Keywords:** protein–protein interactions, redox, cystathionine-β-synthase (CBS)-domains, thioredoxin, intrinsically unstructured (disordered) protein

## Abstract

CP12 is a small, redox-sensitive protein, representatives of which are found in most photosynthetic organisms, including cyanobacteria, diatoms, red and green algae, and higher plants. The only clearly defined function for CP12 in any organism is in the thioredoxin-mediated regulation of the Calvin–Benson cycle. CP12 mediates the formation of a complex between glyceraldehyde-3-phosphate dehydrogenase (GAPDH) and phosphoribulokinase (PRK) in response to changes in light intensity. Under low light, the formation of the GAPDH/PRK/CP12 complex results in a reduction in the activity of both PRK and GAPDH and, under high light conditions, thioredoxin mediates the disassociation of the complex resulting in an increase in both GAPDH and PRK activity. Although the role of CP12 in the redox-mediated formation of the GAPDH/PRK/CP12 multiprotein complex has been clearly demonstrated, a number of studies now provide evidence that the CP12 proteins may play a wider role. In *Arabidopsis thaliana* CP12 is expressed in a range of tissue including roots, flowers, and seeds and antisense suppression of tobacco CP12 disrupts metabolism and impacts on growth and development. Furthermore, in addition to the higher plant genomes which encode up to three forms of CP12, analysis of cyanobacterial genomes has revealed that, not only are there multiple forms of the CP12 protein, but that in these organisms CP12 is also found fused to cystathionine-β-synthase domain containing proteins. In this review we present the latest information on the CP12 protein family and explore the possibility that CP12 proteins form part of a redox-mediated metabolic switch, allowing organisms to respond to rapid changes in the external environment.

## INTRODUCTION

Redox-mediated modulation of enzyme activity is an important post-translational mechanism involved in the regulation of cellular processes, enabling organisms to respond to changes in metabolic demands and environmental conditions. A group of well known redox-sensitive proteins, thioredoxins, play a major role in the regulation of cellular processes in plants, algae and cyanobacteria (Meyer et al., 2009; Buchanan et al., 2012). The mode of action of thioredoxin-mediated regulation is through the post-translational modification of cysteine residues on target proteins, bringing about the conversion of a disulphide bridge in the oxidized state, to a thiol group when reduced. In higher plants two thioredoxins (Trx f and Trx m) were first identified in the 1970s as activators of enzymes involved in photosynthetic carbon assimilation in the chloroplast (Buchanan, 1980; Buchanan and Balmer, 2005). The Calvin–Benson cycle is directly dependent on the energy adenosine triphosphate (ATP) and reducing power nicotinamide adenine dinucleotide phosphate (NADPH) derived from photosynthetic electron transport to drive the enzymatic reactions. In addition, reducing equivalents from electron transport are used to reduce thioredoxin via ferredoxin thioredoxin reductase. Trx f activates the Calvin–Benson cycle enzymes phosphoribulokinase (PRK), NADP-glyceraldehyde-3-phosphate dehydrogenase (GAPDH), fructose 1, 6-bisphosphatase (FBPase) and sedoheptulose 1, 7-bisphosphatase (SBPase) (**Figure 4**). Light intensity in the natural environment is variable and Trx redox state links the activity of these enzymes to the supply of ATP and NADPH in response to variations in light intensity. However, when temperature or light levels fall, the responses of thioredoxin-modulated enzymes in leaf tissues are not uniform. Under such conditions the activities of FBPase and SBPase can temporarily limit photosynthesis (Sassenrath-Cole et al., 1994; Hutchison et al., 2000) however, for PRK and GAPDH no such limitation has been reported and activation is rapid.

A second redox-mediated mechanism that regulates the activity of the Calvin–Benson cycle involves the aggregation of the enzymes PRK and GAPDH into a multiprotein complex which has been shown to be mediated by a small, nuclear-encoded chloroplast protein, CP12. This PRK/GAPDH/CP12 protein complex has been shown to be present in several higher plant (Wedel et al., 1997; Wedel and Soll, 1998; Scheibe et al., 2002; Howard et al., 2011a) and algal species (Avilan et al., 1997; Boggetto et al., 2007; Oesterhelt et al., 2007). The existence of this PRK/GAPDH/CP12 regulatory complex is well established (Avilan et al., 1997; Wedel et al., 1997; Scheibe et al., 2002; Marri et al., 2005b, 2009; Howard et al., 2011a) and when bound in this complex, the activity of the enzymes PRK and GAPDH are decreased. Initially, evidence suggested that the association and dissociation of the PRK/GAPDH/CP12 complex is mediated via changes in NADP(H)/NAD(H) ratios within the chloroplast (Wedel et al., 1997; Wedel and Soll, 1998; Tamoi et al., 2005; Trost et al., 2006). However, more recently it has been shown that the status of the PRK/GAPDH/CP12 complex is regulated by changes in the redox state of Trx (Howard et al., 2008; Marri et al., 2009). *In vitro*, both chloroplastic Trx f and m have been shown to mediate the breakdown of the PRK/GAPDH/CP12 complex, via reduction of the two cysteine pairs on the CP12 protein (Marri et al., 2009). These studies provide evidence of a link between the redox state of Trx and that of CP12 in the formation and breakdown of the PRK/GAPDH/CP12 complex. When high levels of reduced Trx are available, CP12 will be maintained in a reduced state and little or no formation of the PRK/GAPDH/CP12 complex will occur. Conversely when levels of reduced Trx declines, levels of oxidized CP12 will increase resulting in the formation of the PRK/GAPDH/CP12 complex (**Figure 4**).

An important feature of the PRK/GAPDH/CP12 complex *in vivo* is the observation that dissociation and formation of the complex in pea leaves is rapid and that it responds to light intensity (Howard et al., 2008). In high light, dissociation occurred in under 1 min and on transfer to low light re-association was evident within 1 min; furthermore following 5 min in total darkness all of the PRK was found to be associated in the complex. A further important physiological observation is that although PRK contained within the complex is inactive it is found in both the reduced or oxidized state (Lebreton et al., 2003; Howard et al., 2008). The implication of this is that the PRK/GAPDH/CP12 complex provides a mechanism for sequestering and rapidly deactivating PRK and GAPDH in response to reduced light intensity. Conversely when light levels increase PRK and GAPDH are released and the reduced forms do not require Trx-activation and are instantly functional. This may provide an explanation for the rapid increase in PRK activity in response to an increase in light intensity, which is at least one order of magnitude quicker than the rate of Trx-mediated reductive activation of the oxidized form of this enzyme (Avilan et al., 2000). The physiological significance of the PRK/GAPDH/CP12 complex is that it provides a rapid response mechanism to regulate the rate of carbon fixation in the Calvin–Benson cycle, in response to changes in the availability of light to produce NADPH and ATP.

Despite a considerable body of data on the role of CP12 in the context of the regulation of PRK and GAPDH, some questions remain to be addressed in terms of the relative importance of this complex in regulating carbon metabolism and the possibility of a wider role for CP12 in redox regulation of metabolism. Firstly, it is still debatable whether the PRK/GAPDH/CP12 complex is a universal regulatory mechanism. Recent evidence has shown that the CP12 mediated regulation of PRK and GAPDH varies between different algal species (Maberly et al., 2010) and that there is heterogeneity in the PRK and GAPDH protein complex in higher plant species (Howard et al., 2011a). Furthermore, *in vitro* studies indicated that only fully oxidized *Arabidopsis* PRK is incorporated into the complex (Marri et al., 2005b) unlike the findings for the complex isolated from pea and *Chlamydomonas* (Lebreton et al., 2003; Howard et al., 2008). The importance of both CP12 and protein aggregation in the regulation of the Calvin–Benson cycle may therefore vary between species.

Four additional pieces of information raise further questions about the role of CP12. (1) In higher plants the CP12 proteins are encoded by a small gene family with different patterns of expression (Marri et al., 2005a; Singh et al., 2008). (2) Antisense suppression of CP12 in tobacco plants which resulted in a complex phenotype is not consistent with a loss of regulation of the Calvin–Benson cycle (Howard et al., 2011b,c). (3) Recent analysis of genome data from 126 species of cyanobacteria has revealed a wide diversity of CP12 protein sequences raising questions about the role of these different CP12 and CP12-like proteins (Stanley et al., 2013). (4) CP12 is a member of a class of proteins known as intrinsically unstructured proteins (IUPs; Gardebien et al., 2006; Erales et al., 2009b; Mileo et al., 2013). This article presents brings together information from a number of recent studies that suggest that CP12 may have a broader role in the regulation of metabolism, over and above the well established role of CP12 in the regulation of the Calvin–Benson cycle.

### CP12 DISTRIBUTION AND STRUCTURE

**FIGURE 1:**
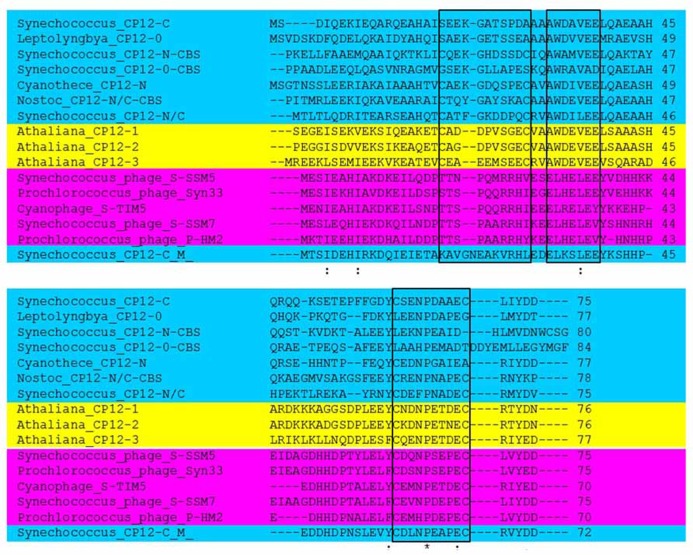
**Sequence analysis comparison of representative CP12 proteins.** CP12 proteins from *Arabidopsis thaliana* (yellow)*,* cyanobacterial CP12 sequences representing the eight different forms (blue) and representative CP12 sequences for cyanophages (pink). Conserved amino acid residues are denoted by (*); conservative changes (:) and semi-conservative (.); black boxes indicate the N- and C-terminal cysteine pairs and the AWD_VEE region. For the *A. thaliana* sequences only the mature proteins are shown and for the CP12-CBS proteins only the CP12-like region is presented. *Arabidopsis* sequences are: AT2G47400 (CP12-1), AT3G62410 (CP12-2), and AT1G76560 (CP12-3). NCBI Reference Sequence for Cyanophages: YP_004324623.1 (*Synechococcus* phage S-SSM5), YP_004323628.1 (*Prochlorococcus* phage Syn33), YP_007006052.1 (Cyanophage S-TIM5), YP_004324068.1 (*Synechococcus* phage S-SSM7), and YP_004323596.1 (*Prochlorococcus* phage P-HM2). Cyanobacterial CP12 sequence’s IMG Gene ID (http://img.jgi.doe.gov/cgi-bin/w/main.cgi?section=FindGenes&page=geneSearch) are: 637616925 (*Synechococcus*_CP12-N/C), 638958550 (*Synechococcus*_CP12-C(M)), 637616815 (*Synechococcus*_CP12-C), 643587017 (Cyanothece_CP12-N), 2509777734 (Leptolyngbya_CP12-0), 2503740304 (Nostoc_CP12-N/C-CBS), 641610209 (*Synechococcus*_CP12-N-CBS), 2506746062 (*Synechococcus*_CP12-0-CBS).

Until recently the distribution of the CP12 proteins had been found exclusively within photosynthetic organisms and at least one CP12-like protein has been identified in all photosynthetic autotrophs including cyanobacteria, with the exception of the prasinophyte *Osterococcus* (Wedel et al., 1997; Wedel and Soll, 1998; Graciet et al., 2003; Marri et al., 2005a; Tamoi et al., 2005; Oesterhelt et al., 2007; Robbens et al., 2007; Groben et al., 2010; Stanley et al., 2013). More recently, it has been shown that proteins containing sequences with a high degree of similarity to the carboxy terminal region of CP12 have been identified in cyanophages (**Figure 1**).

In the *Arabidopsis* genome three genes have been identified and named *CP12-1* (At2g47400), *CP12-2* (At3g62410), and *CP12-3* (At1g76560). CP12-1 and CP12-2 are highly homologous and share 86% identity rising to 98% following cleavage of the transit peptide (**Figure 1**). Comparisons between these proteins in a variety of species have been unable to differentiate CP12-1 and CP12-2 into two separate *sub-*groups on the basis of their amino acid sequence (Singh et al., 2008; Groben et al., 2010). CP12-3 shares 41% and 48% identity with CP12-1 and CP12-2 respectively and phylogenetic analysis places *CP12-3* in a distinct clade. In all angiosperm species for which a full genome sequence is available, e.g., rice, maize and poplar, three CP12 encoding genes have been found with two being highly similar and a third being distinct, similar to the pattern in *Arabidopsis*. In contrast, in the green algae *Chlamydomonas reinhardtii* only one “canonical CP12” coding gene has been identified (Groben et al., 2010; Gontero and Maberly, 2012). In Gymnosperms, similar to *C. reinhardtii*, only one type of CP12 protein has been identified and this has more similarity to the CP12-3-like type. Furthermore, evidence to date suggests that the CP12-1/CP12-2-like forms are not present in this group of plants (Groben et al., 2010).

**FIGURE 2:**
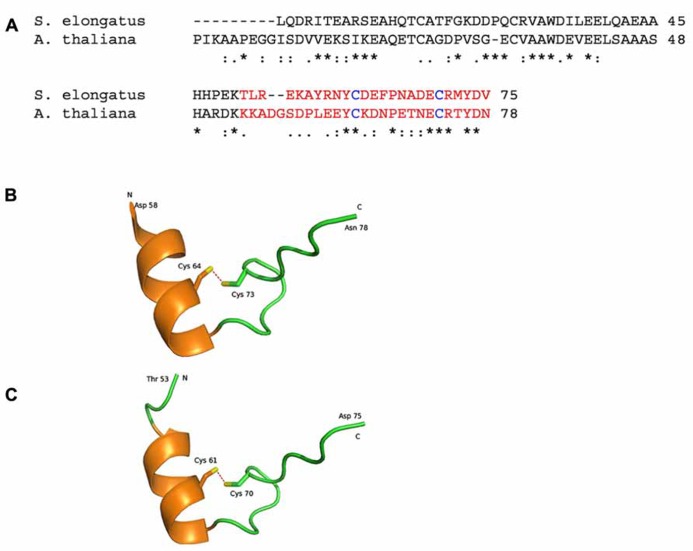
**Representations of crystal structures of the *Arabidopsis thaliana and Synechococcus elongatus* proteins.** **(A)** The amino acid sequence alignment of Q9LZP9 *Arabidopsis thaliana* CP12-1 (mature protein) and Q31RN5 *S. elongatus* (strain PCC 7942). The regions in red is represented in the model structures in (**B**, **C)**. (**B)** 2.0 Å resolution crystal structure of the C-terminal region of CP12 from *Arabidopsis thaliana* (Fermani et al., 2012; PDB accession code 3qv1). **(C).** 2.2 Å resolution crystal structure of the C-terminal region of CP12 from *S. elongatus* (Matsumura et al., 2011; PDB accession code 3b1j). Each structure contains a single alpha helix and a disulphide bond (red dashed lines). N and C represent the ends of the structured region, with the remaining amino acids being disordered and not modeled. Conserved amino acid residues are denoted by (*); conservative changes (:) and semi-conservative (.).

CP12 proteins in the green lineage have a highly conserved primary structure with three key features: an N-terminal cysteine pair, a C-terminal cysteine pair and a core “AWD_VEE” sequence (**Figures 1** and **2**). The N- and C-terminal cysteine pairs have been shown to form two intramolecular disulfide bridges when oxidized which are converted to thiol groups when reduced by Trx. Early studies provided evidence that both the N- and C-terminal disulfide bridges are necessary for the formation of the GAPDH/CP12/PRK complex (Wedel and Soll, 1998; Graciet et al., 2003). Although in the higher plants all CP12 proteins studied so far share all three of these features, there are exceptions to this in the red algae, haptophytes, cyanobacteria, and cyanophages (**Figure 1**; Groben et al., 2010; Thompson et al., 2011; Stanley et al., 2013). An unexpected diversity in the primary structure of CP12-like proteins was found to be present in cyanobacterial species. These have been classified into eight different groups based on the presence or absence of the three conserved features of classical CP12 proteins, i.e., the N- and C- terminal cysteine pairs and the central highly conserved “AWD_VEE” motif (Stanley et al., 2013). In addition, three of the cyanobacterial CP12-like protein classes described have a N-terminal cystathionine-β-synthase (CBS) domain (Stanley et al., 2013). No individual species of cyanobacteria has all eight of the different classes of CP12-like proteins, but with the exception of the marine picoplanktonic group, all other groups have at least one copy of the classical CP12 (CP12 C–N) form.

Structural and *in silico* studies have demonstrated that CP12 has physicochemical properties similar to those of IUPs (Graciet et al., 2003; Gardebien et al., 2006; Erales et al., 2009b; Marri et al., 2010; Mileo et al., 2013). IUPs possess relatively little structure *in vivo*. Instead, they adopt more structured conformations upon binding their target ligand. IUPs (and IUP regions within proteins) typically facilitate protein–protein interactions (Uversky, 2002; Tompa, 2005; Tompa et al., 2005; Uversky et al., 2005). It has therefore been proposed that CP12 is a conditionally unstructured protein and in the reduced state CP12 is disordered and inactive. But under oxidizing conditions, the formation of disulphide bridges and a α-helice results in a more structured active protein. *In silico* modeling of *C. reinhardtii* CP12 predicts the presence of two α-helices located in the N-terminal and central regions of the protein (Gardebien et al., 2006). In contrast, structural studies of cyanobacterial and higher plant GAPDH/CP12 binary complexes reveal only one alpha helix in the C-terminal region (**Figure 2**) while no structure in the N-terminal region of either of these forms CP12 was evident, indicating that this region is highly unstructured (Matsumura et al., 2011; Fermani et al., 2012). On conversion to the reduced form CP12 loses the conserved α-helices present and becomes completely unstructured and this fully unfolded form is more flexible and mobile than oxidized CP12 (Gardebien et al., 2006; Gontero and Avilan, 2011; Matsumura et al., 2011; Fermani et al., 2012). The degree of disorder of CP12 has been shown to increase in higher plants compared to eukaryotic algae and cyanobacteria (apart from the green algal class Mesostigmatophyceae, which is ancestral to the streptophytes) and this has led to the suggestion that CP12 has evolved to become more flexible. This increasing disorder is likely to affect the functionality of CP12 and, given that higher flexibility has been found in other protein to be associated with a wider range of targets, CP12 proteins may have evolved additional roles in higher plants (Groben et al., 2010; Marri et al., 2010).

Bioinformatic analysis of CP12 proteins sequences has revealed some structural similarity with copper chaperones from *Arabidopsis* which have been shown to play different roles in copper homoeostasis (Himelblau et al., 1998; Mira et al., 2001a,b; Delobel et al., 2005). Furthermore, metal binding studies *in vitro* have shown that *Chlamydomonas* CP12 is able to bind both nickel (Ni^2^^+^) and copper (Cu^2^^+^) ions. Although the affinity for nickel is low (Kd 11 μM), the affinity for copper (Kd 26 μM) is within the same range of those reported for the prion protein (Kd of about 14 μM) and for copper chaperone proteins (Multhaup et al., 2001; Cobine et al., 2002; Delobel et al., 2005; Erales et al., 2009a). There is evidence showing that copper ions aid the formation of disulfide bonds in reduced CP12 leading to the recovery of fully oxidized CP12 which led to the hypothesis that the role of CP12 may be linked to copper metabolism (Delobel et al., 2005; Gontero and Maberly, 2012). However, structural studies have shown that GAPDH and PRK can interact with CP12 in the presence or absence of copper ions. In addition, the backbone structures of the GAPDH-CP12 binary complex of *Synechococcus elongatus* in copper-free and copper-bound forms are basically the same suggesting that copper is not essential for CP12 function in relation to the GAPDH/PRK/CP12 complex (Erales et al., 2009b; Matsumura et al., 2011).

### CP12 GENE EXPRESSION

The three CP12 genes present in *Arabidopsis* are differentially expressed (Marri et al., 2005b; Singh et al., 2008). The expression of *CP12-2*, like *GAPDH* and *PRK*, is light dependent and is highest in photosynthetic tissues such as cotyledons, vegetative leaves and stalks. *CP12-1* transcripts are evident in dark-grown tissue and whilst it is abundantly expressed in photosynthetic tissues, it is also expressed in a range of tissues including flowers (siliques, styles, and sepals), seeds and root tips. In contrast *CP12-3* has very low expression in leaf tissue but accumulates in roots, stigma and anthers (Singh et al., 2008). Hypoxic conditions increase expression of *CP12-3* in the leaves while inhibiting the expression of *CP12-2*. Other environmental signals that affect the expression of the CP12 genes include low temperature, which decreases expression of *CP12-2* (*CP12-1* and *CP12-3* are insensitive to this treatment).

**FIGURE 3:**
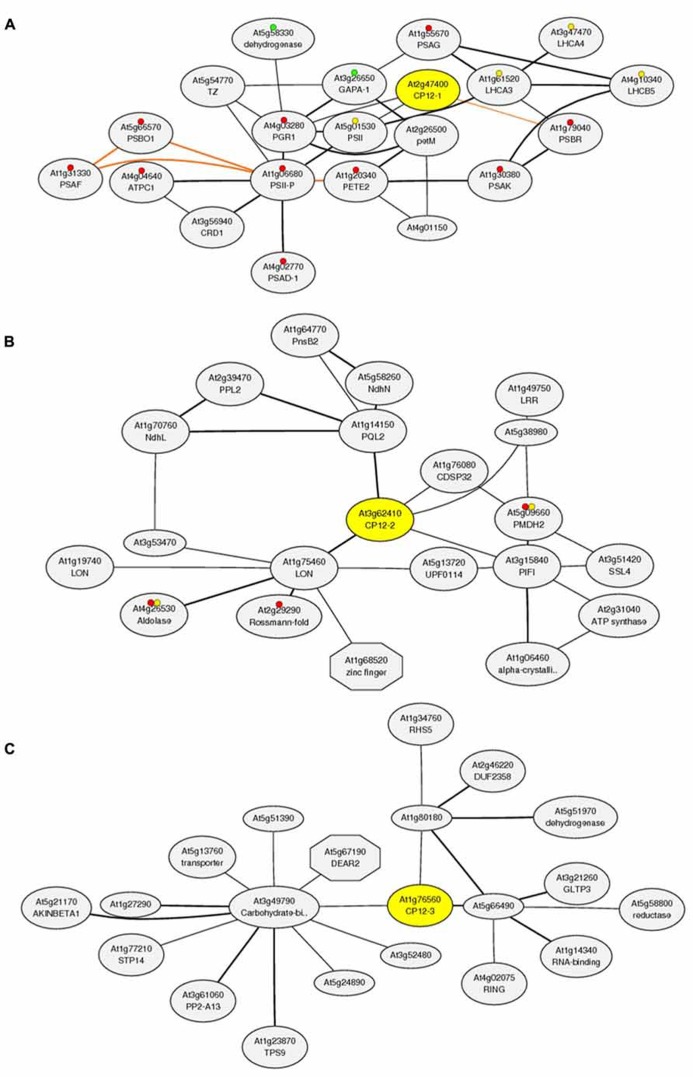
**Co-expression analysis of the CP12 gene family using the *Arabidopsis thaliana* trans-factor and cis-element prediction database, ATTED-II (produced 15/11/2013).** Co-expressed gene network around **(A)** CP12-1 (http://atted.jp/data/locus/819353.shtml). **(B)** CP12-2 (http://atted.jp/data/locus/825414.shtml) and **(C**) CP12-3 (http://atted.jp/data/locus/843989.shtml). Colored marks accompanying some genes represent their involvement in: **(A) Red**: Photosynthesis, **Yellow**: Antenna proteins and **Green**: Carbon fixation in photosynthetic organisms. **(B) Red**: Biosynthesis of secondary metabolites and **Yellow**: Carbon fixation in photosynthetic organisms.

In addition to the interesting results shown by the *in-vivo* expression studies, *in silico* co-expression analysis of the *Arabidopsis* gene family using the *Arabidopsis thaliana* trans-factor and cis-element prediction database, ATTED-II (http://atted.jp, Obayashi et al., 2011) has shown that the three CP12 genes have very distinct co-expression patterns and also correlate with expression of genes outside the Calvin–Benson cycle (**Figure 3**). Although this database is subject to updates which result in differences in the networks produced, the results from this bioinformatics package have consistently shown these tendencies. The co-expression network of *CP12-1* includes *GAPA-1* (a gene encoding the A subunit of GAPDH) and genes encoding the photosynthetic electron transport proteins. Interestingly, expression of *CP12-2* did not correlate with any of the genes encoding Calvin–Benson cycle enzymes, including GAPDH and PRK, but instead the co-expression network included the genes encoding subunits of the thylakoid membrane located NADH-dehydrogenase complex. The *CP12-3* gene shared expression patterns with genes encoding enzymes in phenylpropanoid biosynthesis, carbohydrate metabolism, regulatory kinases and transcription factors; but has failed to show co-expression with genes encoding the photosynthetic electron transport proteins or Calvin–Benson cycle components. The meaning and importance of these co-expression patterns is not yet clear and experimental approaches will be needed to understand the specific implications of these connections. Nevertheless, the differences in the co-expression patterns of the three plant CP12 genes raise questions about the role of the different CP12 isoforms and about the influence of CP12 regulation in wider metabolism.

### CP12 *IN VIVO* FUNCTION

To date two studies have reported on the effects of reduced levels of CP12 protein *in vivo*. One using the cyanobacterial knock out mutant of *Synechococcus* PCC7942 (Tamoi et al., 2005) and the other tobacco antisense CP12 plants (Howard et al., 2011b,c). The resulting phenotypes indicate that CP12 has an important role in the regulation of metabolism. In *Synechococcus* PCC7942 the results were consistent with the proposal that CP12 was necessary for the separation of the activity of the Calvin–Benson cycle from the oxidative pentose phosphate pathway (OPPP) during day-night cycles. As a corollary to this study it was recently reported that cyanobacterial phage exploit this regulatory mechanism by introducing a copy of a CP12-like protein into the cyanobacterial host, which results in a down regulation of the Calvin cycle and an increase in flux through the OPPP. In addition to expression of a CP12 gene, phage genes involved in the light reactions, deoxynucleotide biosynthesis, and the OPPP, including a transaldolase gene, are all expressed in the host cyanobacteria. It is proposed that the cyanophage uses this metabolic switching strategy to boost production of NADPH to help fuel the biosynthesis of deoxynucleotides for phage replication (Thompson et al., 2011).

The situation in higher plants is likely to be different to that found in cyanobacteria. In higher plants not only are some Calvin–Benson cycle enzymes reductively activated in the light, but Trx f also reduces plastidic glucose 6-phosphate dehydrogenase (G6PDH), the first enzyme of the OPPP, which results in the inactivation of G6PDH (Wenderoth et al., 1997; Kruger and von Schaewen, 2003; Nee et al., 2009). Such a mechanism could be sufficient to prevent futile cycling between the Calvin–Benson cycle and the OPPP. Analysis of flux into the OPPP, measured by following the decarboxylation of 6-phosphogluconate to ribulose 5-phosphate catalyzed by 6-phosphogluconate dehydrogenase, together with measurements of G6PDH activity in antisense CP12 plants suggested that the cyanobacterial model cannot be applied to tobacco (Howard et al., 2011b). Furthermore, antisense suppression of CP12 had a limited effect on the ability of the PRK/GAPDH/CP12 complex to form in the presence of NAD and no significant impact on PRK or GAPDH enzyme activity or photosynthetic carbon fixation was detected. In contrast to this, significant changes in the growth rate and very dramatic alterations to morphology were observed in the CP12 antisense plants including a loss of apical dominance, fused cotyledons, altered leaf morphology and reduced fertility (Raines and Paul, 2006; Howard et al., 2011b,c). Furthermore, carbon allocation to the cell wall increase with a concomitant decrease in allocation of carbon to starch and soluble carbohydrates. Interestingly, in the CP12 antisense plants the activity of the Trx–activated enzyme NADP-malate dehydrogenase (NADP-MDH) was lower than in wild type plants and changes in pyridine nucleotide content were evident, suggesting a reduction in the activity of the malate valve. This observation is made more interesting because this decrease in NADP-MDH activity corresponded to changes in mobility of this enzyme analyzed using Blue Native PAGE (Howard et al., 2011b). These results indicated a structural change in NADP-MDH which could be due either to a change in conformation or aggregation state of this enzyme. Activation of NADP-MDH involves conformational changes and is subject to a two-stage process mediated by reduced Trx m. The change in mobility of this enzyme together with reduced activity in the antisense CP12 plants suggests that either CP12 is required for activation of NADP-MDH, or that the consequences of loss of CP12 in the plants impacts indirectly on NADP-MDH (Scheibe, 2004; Scheibe et al., 2005; Schneidereit et al., 2006; Howard et al., 2011b). Any reduction in NADP-MDH activity would be expected to impact on the ability to dissipate excess NADPH through the conversion of oxaloacetate to malate, via the malate valve (Scheibe, 2004; Scheibe et al., 2005). Interestingly, in the CP12 antisense plants significant reductions in levels of both 2-OG and malate were observed, indicating that there may have been impairment of the malate valve which in turn negatively affected 2OG cycling (Howard et al., 2011b). This result is consistent with the proposal that the AtpOMT1 transporter has a dual function in OAA/malate exchange in the malate valve and in 2-OG/malate exchange for carbon/nitrogen metabolism (Riebeseel et al., 2010).

### CP12-LIKE FUSION PROTEINS

In *C. reinhardtii* the GAPDH holoenzyme is a homotetramer made up of A-type subunits and it has been shown that CP12 binding to this form of GAPDH confers redox regulation, mediated directly by Trx (Graciet et al., 2003). In higher plants there is also a B form of the GAPDH subunit which forms a functional heterotetramer (A2B2) with the A subunit. The B subunit of GAPDH found in higher plants is believed to have arisen due to a gene duplication of the GapA gene and a subsequent fusion with the C-terminus of CP12 (Pohlmeyer et al., 1996; Petersen et al., 2006). This C-terminal extension contains two cysteine residues and has been shown to confer Trx-mediated redox regulation on the GAPDH A2B2 enzyme (Sparla et al., 2002). It had been suggested that CP12 provides redox regulation to the A4 homotetramer but that the A2B2 form is autoregulated (Trost et al., 2006). However, the picture is now less clear as CP12 has been identified as a component of a complex containing the A2B2 heterotetramer (Carmo-Silva et al., 2011). Furthermore higher plants species with no detectable A4 homotetramer have been shown to form a PRK/GAPDH/CP12 complex *in vitro* (Wedel et al., 1997; Scheibe et al., 2002; Howard et al., 2008; Carmo-Silva et al., 2011). More recently a second group of CP12 fusion proteins has been identified from analysis of cyanobacterial genomes. In addition to the diversity of classes of CP12 protein it has been shown that some CP12-like proteins exist as fusions with proteins containing a CBS domain (Stanley et al., 2013). The function of these CBS domain-containing proteins has not been defined and the role of the CP12 fusion in relation to the activity of this domain also remains to be resolved. Having said this, it is interesting to speculate that the fusion of the CP12-like motifs to the CBS domain containing proteins in cyanobacteria confers a redox regulation to the activity of the CBS protein, similar to that for GAPDH in higher plants.

### A CBS-TRX-CP12 REDOX NETWORK?

**FIGURE 4:**
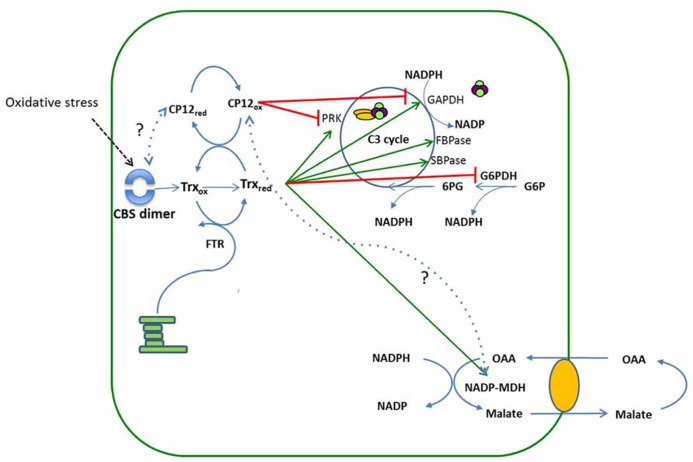
**A schematic showing the relationship between CBS domain containing proteins, thioredoxin and CP12 regulation of the Calvin cycle.** The reducing equivalents from the electron transport chain reduce the disulphide bridges on thioredoxin to thiol groups. Reduced Trx then reductively activates enzymes of the Calvin–Benson cycle, PRK, GAPDH, SBPase, FBPase and the enzyme MDH. The redox state of CP12 is also determined by Trx, when light levels are high Trx is maintained in a reduced state and under these conditions CP12 is reduced. When light levels drop, Trx becomes oxidized and the levels of CP12 in the oxidized state also increase resulting in the formation of the PRK/GAPDH/CP12 complex and inactivation of PRK and GAPDH. Under oxidative stress dimerization of the plastid CBSX1 protein occurs, which increases the levels of reduced Trx, thereby maintaining CP12 in the reduced state. The green arrows indicate the Trx mediated activation of the enzymes GAPDH, PRK, SBPase, FBPase, and MDH and the red bars indicate deactivation of PRK and GAPDH by CP12 mediated formation of the complex or of G6PDH by reduced Trx. Dotted lines indicate hypothetical interactions between CBSX1 and CP12red and CP12ox and MDH.

The discovery of eight different classes of CP12 in cyanobacteria, some of which are fused to a CBS domain containing protein, raises interesting questions about the role of these fusion proteins in the regulation of metabolism. Evidence from *in silico* modeling studies indicates that the CBS-CP12 fusion proteins are unable to interact with GAPDH and therefore an alternative role for these proteins has to be considered (Stanley et al., 2013). In higher plants, although a large family of CBS domain type proteins has been identified, none have been found fused to a CP12-like domain (Kushwaha et al., 2009). However, two CBS domain-containing proteins in *Arabidopsis*, CBSX1 and 2 have been shown to be located in the chloroplast. Analysis of the CBSX1 and 2 insertion mutants revealed that theses proteins form a dimer under oxidative stress conditions and that in these mutants the level of reduced Trx f and m was increased (Yoo et al., 2011). This in turn will maintain a higher level of the reduced form of CP12, thereby modulating the activity of the Calvin–Benson cycle (**Figure 4**). CP12 may also influence directly the ability of the CBSX proteins to dimerize in response to changes in redox state in the chloroplast through Trx action on CP12 (**Figure 4**). Although there is no direct evidence to support this, it is worthy of consideration given the presence of CP12 fusions with CBS domain containing proteins in cyanobacteria.

## CONCLUSION

As yet no experimental evidence for a role for the CP12 proteins outside of the Calvin–Benson cycle has been shown nor has a unique function been assigned to the different forms of CP12 in any organism. However, evidence from a number of different sources is accumulating to suggest that the CP12-like proteins may act in combination with other regulatory proteins, e.g., Trx’s and CBS domain containing proteins to modulate metabolism in response to changes in metabolic demand and environment, mediated by changes in the redox state. This raises the possibility that CP12 acts to switch on and off metabolic pathways in response to changes in redox status in the chloroplast network.

## Conflict of Interest Statement

The authors declare that the research was conducted in the absence of any commercial or financial relationships that could be construed as a potential conflict of interest.
